# Molecular Vibration Explorer: an Online Database and Toolbox for Surface-Enhanced Frequency Conversion and Infrared and Raman Spectroscopy

**DOI:** 10.1021/acs.jpca.2c03700

**Published:** 2022-07-06

**Authors:** Zsuzsanna Koczor-Benda, Philippe Roelli, Christophe Galland, Edina Rosta

**Affiliations:** † Department of Physics and Astronomy, University College London, London, WC1E 6BT, United Kingdom; ‡ Department of Chemistry, King’s College London, London, SE1 1DB, United Kingdom; § Nano-Optics Group, CIC nanoGUNE BRTA, 20018 San Sebastián, Spain; ∥ Institute of Physics, Ecole Polytechnique Fédérale de Lausanne (EPFL), CH-1015 Lausanne, Switzerland

## Abstract

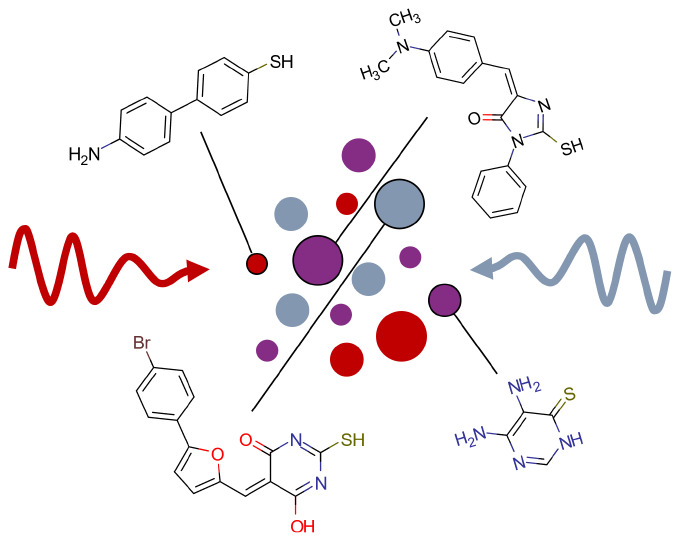

We present Molecular Vibration Explorer, a freely accessible online database and interactive tool for exploring vibrational spectra and tensorial light-vibration coupling strengths of a large collection of thiolated molecules. The “Gold” version of the database gathers the results from density functional theory calculations on 2800 commercially available thiol compounds linked to a gold atom, with the main motivation to screen the best molecules for THz and mid-infrared to visible upconversion. Additionally, the “Thiol” version of the database contains results for 1900 unbound thiolated compounds. They both provide access to a comprehensive set of computed spectroscopic parameters for all vibrational modes of all molecules in the database. The user can simultaneously investigate infrared absorption, Raman scattering, and vibrational sum- and difference-frequency generation cross sections. Molecules can be screened for various parameters in custom frequency ranges, such as a large Raman cross-section under a specific molecular orientation, or a large orientation-averaged sum-frequency generation (SFG) efficiency. The user can select polarization vectors for the electromagnetic fields, set the orientation of the molecule, and customize parameters for plotting the corresponding IR, Raman, and sum-frequency spectra. We illustrate the capabilities of this tool with selected applications in the field of surface-enhanced spectroscopy.

## 1 Introduction

Infrared (IR) absorption and Raman scattering spectroscopy constitute the most informative nondestructive optical methods to obtain fingerprints of molecular species. While both signals are intrinsically too weak to allow detection of single molecules or molecular monolayers, surface-enhanced IR and Raman spectroscopy (SEIRA and SERS, respectively) circumvent this limitation by leveraging a combination of chemical and electromagnetic (plasmonic) enhancement factors. The former describe the effects of orbital modifications and charge transfer on IR and Raman activities when a molecule binds to a metal (most often gold, silver, copper, etc.). The latter results from the enhanced local fields provided by localized surface plasmon resonances supported by metallic nanostructures. Over the years, the power of SEIRA and SERS for molecular spectroscopy has been firmly established, with applications extending beyond fundamental research into biosensing and security.^1−3^ IR absorption and Raman scattering can also be leveraged together in a process called vibrational sum-frequency generation,^4^ which has proven useful for time-resolved studies of vibrational properties and dynamics of molecules at interfaces,^5−7^ such as in the context of catalysis. Moreover, recent proposals and experiments inspired by the field of cavity optomechanics^8,9^ have translated this technique into a potential technology for frequency upconversion from the THz and IR domain into the visible domain.^4,10^ Another related and emerging research field is that of vibrational polaritons,^11−13^ which aims in particular at remotely controlling chemical properties and reaction rates through strong coupling of vibrational modes with electromagnetic vacuum fluctuations.

Online tools such as IRCalc^14^ provide valuable insights into molecular vibrations, IR and Raman activity at the empirical or semiempirical level, for mainly educational purposes.

Here, we present an open source online platform called Molecular Vibration Explorer (MVE).^15^ This platform is an interactive molecular library for the design of cutting edge spectroscopic applications, allowing to explore vibrational spectra and light-vibration coupling for integration in plasmonic antennas and cavities. With a database of thousands of commercially available compounds and easy-to-navigate online tools, our work paves the way for fast and uncomplicated molecular design. Indeed, many groups developing these experimental techniques lack hardware, software, time or workforce to search for molecules with optimal properties for a specific goal, including IR vibrational strong coupling, frequency upconversion, Raman tag, and so on.

Our database is based on accurate DFT calculations of molecular vibrational properties that are made openly accessible and is complemented by comprehensive numerical tools for easy exploration, analysis and selection of molecules. Among the main features available on our platform are the following:

- For each molecule, orientation-averaged and orientation-dependent IR, Raman, and frequency conversion (SFG) spectra.
- Interactive sorting and screening of the entire database over user-defined frequency ranges according to a chosen parameter, such as IR or Raman cross-section.
- 3D interactive plots of molecules and their vibrational modes, dynamically linked to the orientation-dependent values of IR and Raman cross sections.
- Identification of chemically similar molecules within the database as well as from a list of known self-assembling molecules.

In the following, we first describe the theory and methodology behind the toolbox (sections 2.1 and 2.2), before presenting its main functionalities in section 3.1. We conclude by discussing examples of potential applications in section 3.2.

## 2 Methods

### 2.1 Definitions

We calculate both orientation-averaged (denoted by angle brackets) and orientation-specific intensities for IR absorption, Raman scattering and frequency conversion (specifically, SFG) processes. The IR absorption intensity (in [km mol^–1^] units) of vibrational mode *m* is defined as
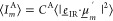
1where *e*_IR_ the field polarization vector of the IR beam, μ_*m*_^′^ is the dipole derivative vector, and *C*^A^ = 2.9254 × 10^3^, assuming μ_*m*_^′^ is given in atomic units [e-bohr·bohr^–1^·amu^–1/2^].

The Raman intensity (differential Raman cross-section) for Stokes bands is given in [cm^2^·sr^–1^] units
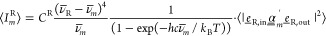
2where ν̅_R_ and ν̅_*m*_ are the wavenumbers of the pump laser and of the normal mode, respectively. The prefactor includes the effect of thermal occupancy of the vibration at temperature *T*. The polarization vectors of the pump (in) and Raman scattered (out) fields are given by *e*_R,in_ and *e*_R,out_, and  is the polarizability derivative tensor. If  is given in [bohr^4^·amu^–1^] units and wavenumbers in [cm^–1^], the constant scaling factor is *C*^R^ = 2.060 × 10^–45^.

The SFG intensity is calculated as
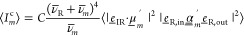
3where *C* = *C*^A^*C*^R^. *I*_*m*_^*c*^ measures the net increase in anti-Stokes Raman intensity due to IR pumping, hence the plus sign in the wavenumber dependent factor and the omission of the Bose Einstein occupancy.

Note that when averaging over multiple orientations, the overlap of the IR and Raman modes need to be considered for each orientation individually, as
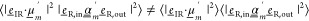
4Nevertheless, analytic formulas for calculating the averages over all possible relative orientations of the molecule and fields, considering all possible field polarization settings, can be derived (cf. Supporting Information, section S1). For orientation-specific intensities, the angle brackets in eqs 1–3 can be omitted.

Apart from individual normal mode intensities, the target properties *A*, *R*, and *P* defined below can also be used for ranking the molecules of the database in a specific frequency range.
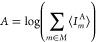
5
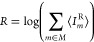
6
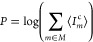
7where *M* refers to the set of normal modes that have frequencies in the range defined by the user. These properties are standardized as described in ref (16). *A*, *R*, and *P* can also be calculated using orientation-specific intensities, this enables fast comparison of molecular performance at different orientations.

The vibrational spectra of each molecule can be plotted as discrete (stick) spectra or broadened spectra. In the latter case, a Lorentzian broadening is applied to IR and Raman spectra, for which fwhm_IR_ and fwhm_R_ can be specified separately, and the SFG spectrum inherits the line shape of the product of these two Lorentzians.

### 2.2 Computational Methods

The creation of the database is described in ref (16). Currently, compounds containing thiols ensuring strong affinity to gold surfaces are explored based on machine learning-predicted and randomly selected molecules. The “Thiol” database consists of unbound thiolated compounds, while the “Gold” database simulates the immediate effects of linkage to the surface by substituting the thiol hydrogen to a gold atom. While the “Gold” database was specifically created for surface-enchanced applications, the “Thiol” database can be used for modeling gas/solution experiments (see, e.g., SI, Figure S5), which are usually performed as a quick first check when screening new molecules. Our databases can be easily extended to include additional readily available compounds from tens of millions of commercially available molecules (from databases such as eMolecules, MolPort, or Sigma-Aldrich). DFT calculations are performed to determine molecular geometries and vibrational properties at the B3LYP+D3/def2-SVP level using the Gaussian program package. This level of modeling was shown to be sufficiently accurate for both the “Thiol” and “Gold” databases when determining the relative intensities of a set of test compounds and simulating the finer details of vibrational spectra, by comparison to solution phase Raman and SERS experiments, respectively.^16^ See also an example in SI, Figure S5. Binding to the metal surface has been studied for a couple of molecules like thiophenol previously. These include modeling of gold clusters^17^ or slabs^18^ and give valuable information on the details of vibrational spectra. According to our investigations (ref (16), SI, Figure S5), the “Gold” database gives a sufficiently good match with SERS experiments to be used in an exploratory manner for surface-enhanced applications. Analytic formulas for the orientation averages of intensities were derived using *Mathematica* and are given in SI, section S1. MVE can be accessed via the Materials Cloud platform. The web application is created using *Voila* and is based on interactive Jupyter notebooks. Screenshots of the web application pages are also shown in SI, section S2. Molecular similarity scores are generated using RDKit. NGLView is used for the 3D visualization of molecules.

## 3 Results and Discussion

### 3.1 Functionalities

#### 3.1.1 Exploring the Databases

The “Gold” and “Thiol” databases can be explored separately, or alternatively, specific molecules can be checked simultaneously in the two databases by using the search tool described in section 3.1.4. The database page allows for ranking molecules within the database and selecting them for further analysis. The user may choose the *target property* (*A*,*R*, or *P*) and set a frequency range of interest. A global frequency scaling factor can be applied to improve agreement between DFT calculated and experimentally measured frequencies. The respective directions of the field polarization vectors (*e*_IR_, *e*_R,in_, and *e*_R,out_) can be specified independently, among three possible orthogonal directions *x*, *y*, and *z*. Intensities ⟨*I*_*m*_^A^⟩, ⟨*I*_*m*_^R^⟩, and ⟨*I*_*m*_^c^⟩ have been precomputed and stored for all possible combinations of field polarizations and considering a random orientation of molecules. A temperature of 298.15 K and Raman laser wavelength of 785 nm were used for computing Raman and conversion intensities. The distribution of the target property across the entire database, for mode frequencies within the user-specified range, is plotted as a histogram, and a table is generated below that shows all molecules sorted according to decreasing value of the target. Further properties of the molecules and their individual normal modes can be explored by navigating the links displayed in the table, as described below.

#### 3.1.2 Molecular Properties

Upon clicking on “*Go to molecule page*” next to a particular molecule, the new page displays vibrational spectra and other relevant molecular properties and enables a high level of customization. In the tab labeled “*Set molecule orientation*” on the left, the molecular orientation can be modified at will by specifying the rotational angles ϕ, θ, and ξ with respect to the Cartesian coordinate system defined along the possible polarization vectors. The resulting orientation is instantly displayed on the 3D molecular viewer while the corresponding Raman, IR, and SFG spectra are also updated. The spectra for the selected orientation can be directly compared with those for the orientation average by checking “*Show full orientation average*” in the “*Set plotting parameters*” tab on the left. For Raman scattering and SFG the temperature and laser wavelength can be set in the “*Set experimental parameters*” tab, while the frequency range, scaling factor, style of the spectrum (stick, broadened, stick+broadened) and corresponding broadening parameters (fwhm_IR_, fwhm_R_) can all be tuned in the “*Set plotting parameters*” tab. Finally, field polarization directions can be specified in the “*Set field polarization*” tab.

In addition to optical properties, physical properties of the molecule relevant to surface-enhanced spectroscopy are listed below the 3D drawing. The length of the projection of the molecule along the *z* axis is referred to as “*Layer height*”. Indeed, it corresponds to the approximate thickness of a molecular monolayer assuming binding through the thiol group onto a flat gold surface spanning the *x* and *y* plane. Its geometrical projection on the flat gold surface is given by “*XY projection*”. These properties can be used for estimating how the enhancement factor of surface-enchanced experiments is affected by the geometrical properties of the molecule, as described in ref (16). Another feature that is especially useful for navigating the database is the table of similar molecules that is displayed at the bottom of the page. Molecules susceptible to form self-assembled monolayers (SAMs) and similar to the chosen molecule are listed to help optimization of surface-enhanced applications.

#### 3.1.3 Properties of Vibrational Modes

Upon clicking on “*Check normal modes*” next to a particular molecule, the user can explore all normal modes of the selected molecule through 3D visualization. The dependence of the IR, Raman, and conversion/SFG intensity of a specific normal mode on molecular orientation is represented by a projection onto the plane perpendicular to the axis of rotation. The direction of the field polarization vectors of the IR and Raman (excitation and scattered) beams can also be set on this page.

#### 3.1.4 Searching the Database

MVE features a SMILES-based search tool that operates with molecular similarity scores. Potential hits and the most similar molecules are listed for the “Thiol” and “Gold” databases, as well as for the library of known SAMs. For molecules that are included in both databases, the search tool provides a quick way to compare properties of thiolated and gold-linked forms (see SI, section S3 for an example). This comparison can be used for example to exploit the effects of surface linkage (chemical enhancement) or conversely to select robust vibrational peaks, not affected by a change in linkage.

### 3.2 Example Applications

**Figure 1:**
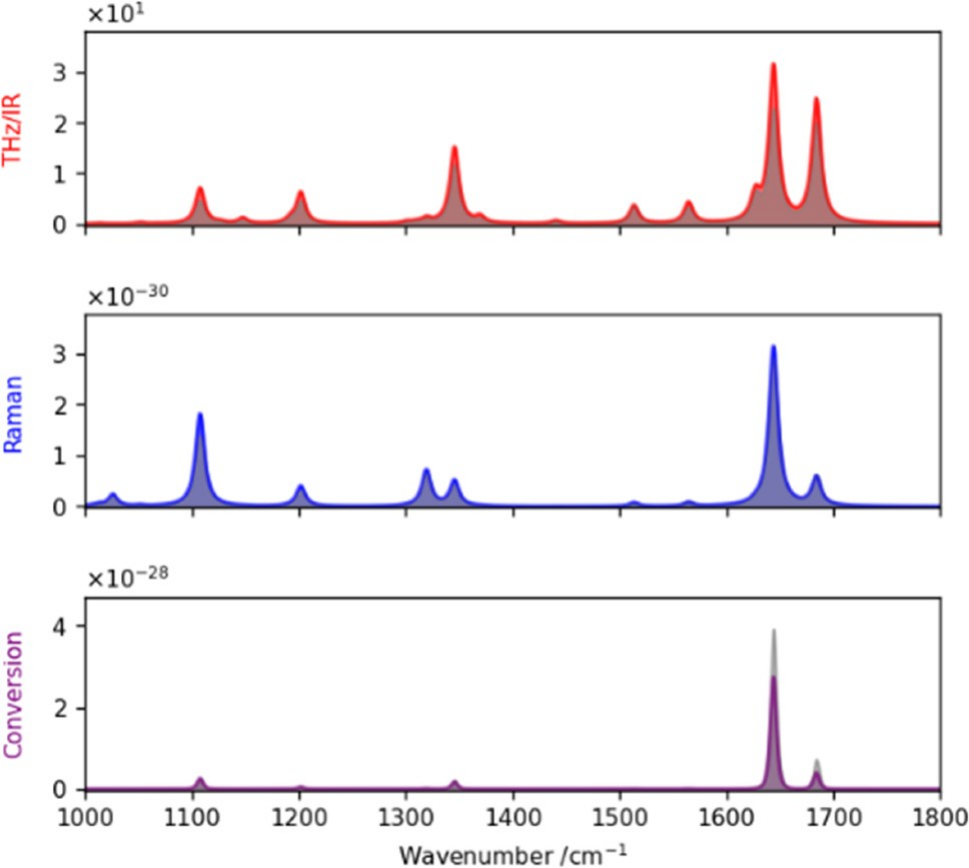
From top to bottom, numerical simulations of IR absorption (red line), Raman scattering (blue), and conversion (SFG, purple) spectra of a NH_2_-BPhT molecule for a specific orientation. The orientation averaged spectra is depicted with gray areas for each spectrum. All local fields are chosen to be parallel to the *z*-axis.

Sum-frequency generation (SFG) has proven to be a useful tool for a variety of investigations in surface science.^5^ The combined selection rules of IR and Raman processes enable to optically study the symmetry of crystalline structures,^19^ to investigate the bonding of molecules to metallic surfaces^20−22^ and the orientation of molecular layers on different substrates.^23,24^ The experimental characterization of these molecules requires access to the nonvanishing elements of the nonlinear polarizability tensor, which can for certain molecules be addressed sequentially using different polarization combinations. All these components can be extracted with the help of our toolbox and can be used to reconstruct the IR, Raman, and upconversion (SFG) spectra for all combinations of local electromagnetic fields and orientation of the molecule. Figure 1 illustrates different spectra obtained for the NH_2_-BPhT molecule both for a specific orientation of the molecule and for the orientation-averaged case. In this section of the manuscript, the specific orientation will correspond to the initial orientation of the “Gold” database’s molecules in MVE: S–Au bond along the *z*-axis, while the polarization of the three local electromagnetic fields (IR, Raman excitation, Raman scattering), if not explicitly given in the text, will be chosen along *z*. This default configuration is expected to well approximate most common situations in surface-enhanced studies.^25^ One interesting feature for spectroscopy is that the more constrained selection rules for SFG allow only a few vibrational modes to feature sizable upconversion efficiency. This aspect can be leveraged in experiments where the level of background arising from the substrates or other molecules present in the vicinity might prevent detection of the targeted molecules.

**Figure 2:**
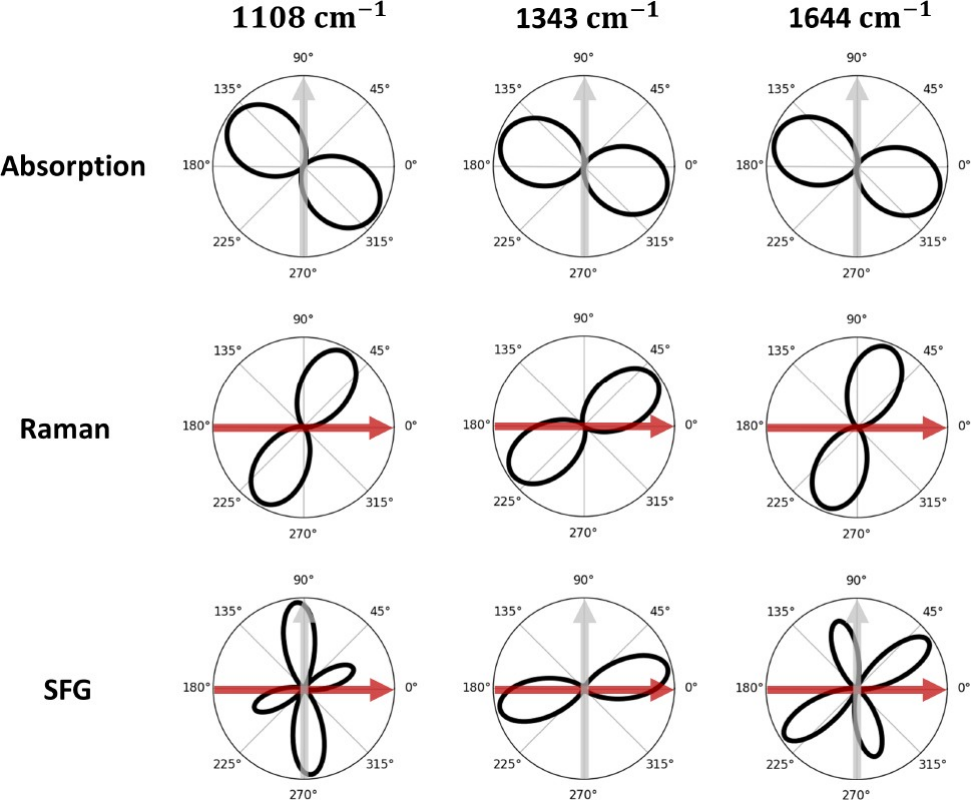
From top to bottom, numerical simulations of the dependence of the IR absorption, Raman scattering, and SFG spectra on the orientation of the molecule with respect to external local fields for the three main upconversion-active modes of NH_2_-BPhT. In this figure, local fields are chosen to be cross-polarized: IR field along *y* (gray arrow) and Raman in/out fields along *x* (red arrow).

In the situation where the polarizations are defined by a plasmonic nanostructure and a specific orientation of the molecule is expected,^4^ the MVE toolbox permits to investigate the orientation dependence of the IR absorption, Raman, and SFG signals. Figure 2 illustrates, for the case of cross-polarized local fields (IR field along *y*, Raman in/out fields along *x*), the changes in the different processes’ magnitudes as a function of the molecule orientation. As evidenced in the figure, even though the corresponding absorption and Raman signatures look rather similar, the molecular orientation dependence of SFG efficiency shows clearly distinct patterns for different vibrational modes of NH_2_-BPhT. We note that a cross-polarization configuration is not typical in doubly resonant plasmonic cavities but could be achieved using specific antennas.^26^ Thanks to the prediction of orientation dependence the MVE toolbox may help to optically assess the orientation of self-assembled monolayers within metallic structures^27^ in a similar way as it could be determined in TERS experiments.^28,29^ The MVE toolbox will also be useful in designing nanostructures and molecular layers maximizing the conversion efficiency of the SFG process for frequency conversion applications.^4,9^

#### 3.2.1 Molecule Optimization or Fingerprinting

**Figure 3:**
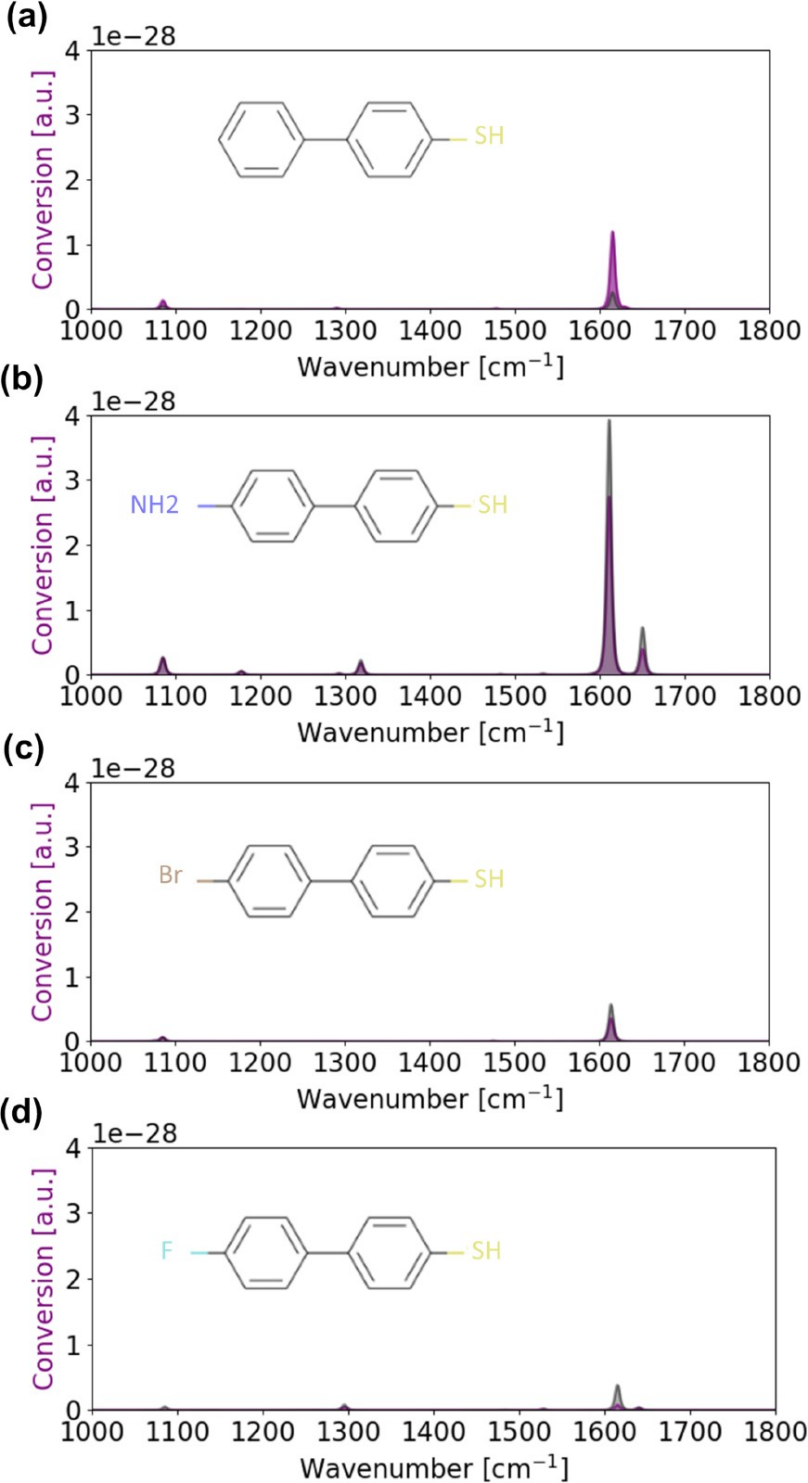
Averaged (gray area) and orientation specific (purple line) conversion spectra of BPhT (a), NH_2_-BPhT (b), Br-BPhT (c), and F-BPhT (d).

Another opportunity offered by the MVE database is to scrutinize molecules similar to a reference molecule already controlled experimentally. As demonstrated in Figure 3, a simple change in one termination of the BPhT molecule can lead to significant changes in the respective magnitude of the different SFG active modes. The toolbox therefore provides a way to optimize upconversion efficiency by carefully designing the molecule and could also be used reversely in order to experimentally differentiate the upconverted signals from very similar molecules, changing only by one termination.^30^

#### 3.2.2 SERS and Advanced IR Detection

**Figure 4:**
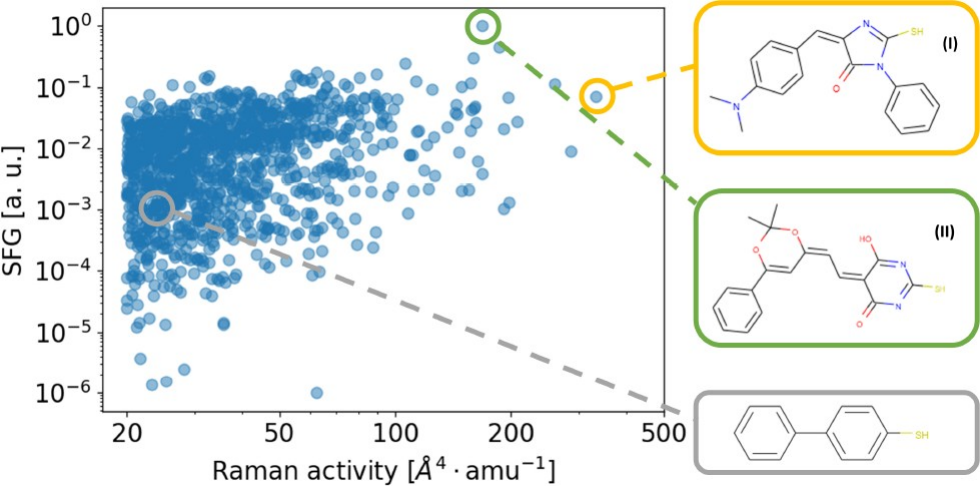
Distribution of SFG coefficients and Raman activities for all vibrational modes of the “Gold” database lying in the 20–50 THz spectral range, identified in ref (9), for its interest in IR detector applications. We highlight BPhT with a gray circle, the only molecule ever used to date in a continuous wave SFG experiment.^4^ Two other molecules with significantly higher calculated SFG efficiencies are highlighted in yellow and green. Full names of the molecules are given in SI, section S4.

One direct application of the MVE database consists in exploring possible molecules for improved SERS signals in applications such as SERS tags^31,32^ or fundamental studies of molecular optomechanics.^8,33^ As evidenced in Figure 4, there are numerous commercially available molecules compatible with gold functionalization that would reach levels of Raman activity comparable or higher than standard compounds used in SERS experiments like BPhT. Additionally, filtering the database on the region of interest for low-light IR detectors at room temperature^9^ (20–50 THz), we can identify molecular vibrations with SFG coefficients orders of magnitude larger than the molecule (BPhT) used in the recent surface-enhanced upconversion experiments.^4,10^

#### 3.2.3 IR Vibrational Strong Coupling

**Figure 5:**
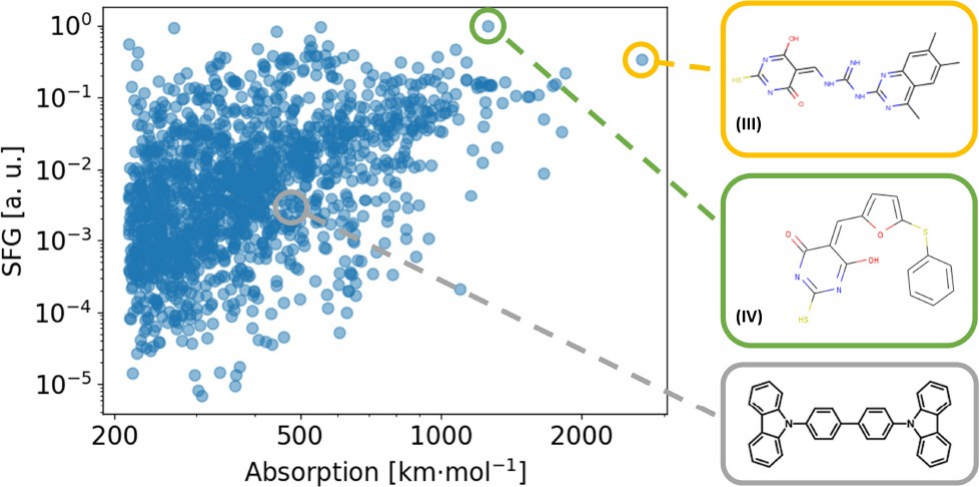
Distribution of SFG coefficients and Raman activities for all vibrational modes of the “Gold” database lying in the 1400–1550 cm^–1^ range that match surface phonon-polariton (SPhP) modes of hexagonal boron nitride (hBN). We highlight with a gray circle the molecule (CBP) experimentally used to achieve vibrational strong coupling with an SPhP mode of hBN.^13^ Two other molecules with significantly higher calculated IR absorption cross sections and SFG efficiencies are highlighted in yellow and green. Full names of the molecules are given in SI, section S4.

The MVE database also opens new routes to explore the regime of vibrational strong coupling (VSC)^12^ in systems containing one or few molecules only. Recent studies of the coupling between molecular vibrations and surface phonon–polaritons modes of hBN have achieved this regime.^13^ The narrow range accessible with these phonon–polaritons modes (1400–1550 cm^–1^) requires very specific molecules to achieve sufficiently strong coupling. MVE enables to quickly identify molecules (Figure 5) with IR cross sections that are promising for strong coupling applications. In addition, the database allows to identify vibrational modes that would not only reach the IR vibrational strong coupling regime but would simultaneously enable substantial conversion of the generated phonons to the visible via molecular optomechanical upconversion. The molecule used in ref (13) is illustrated on the same figure to highlight the number of other molecular vibrations that would achieve similar or better IR intensities while enabling more suitable or controlled deposition, smaller footprint or higher compatibility with molecular SFG experiments. The same approach could also be instrumental in the design of the interaction between other surface modes like graphene plasmons and vibrations.^36,37^

#### 3.2.4 Molecule Detection

**Figure 6:**
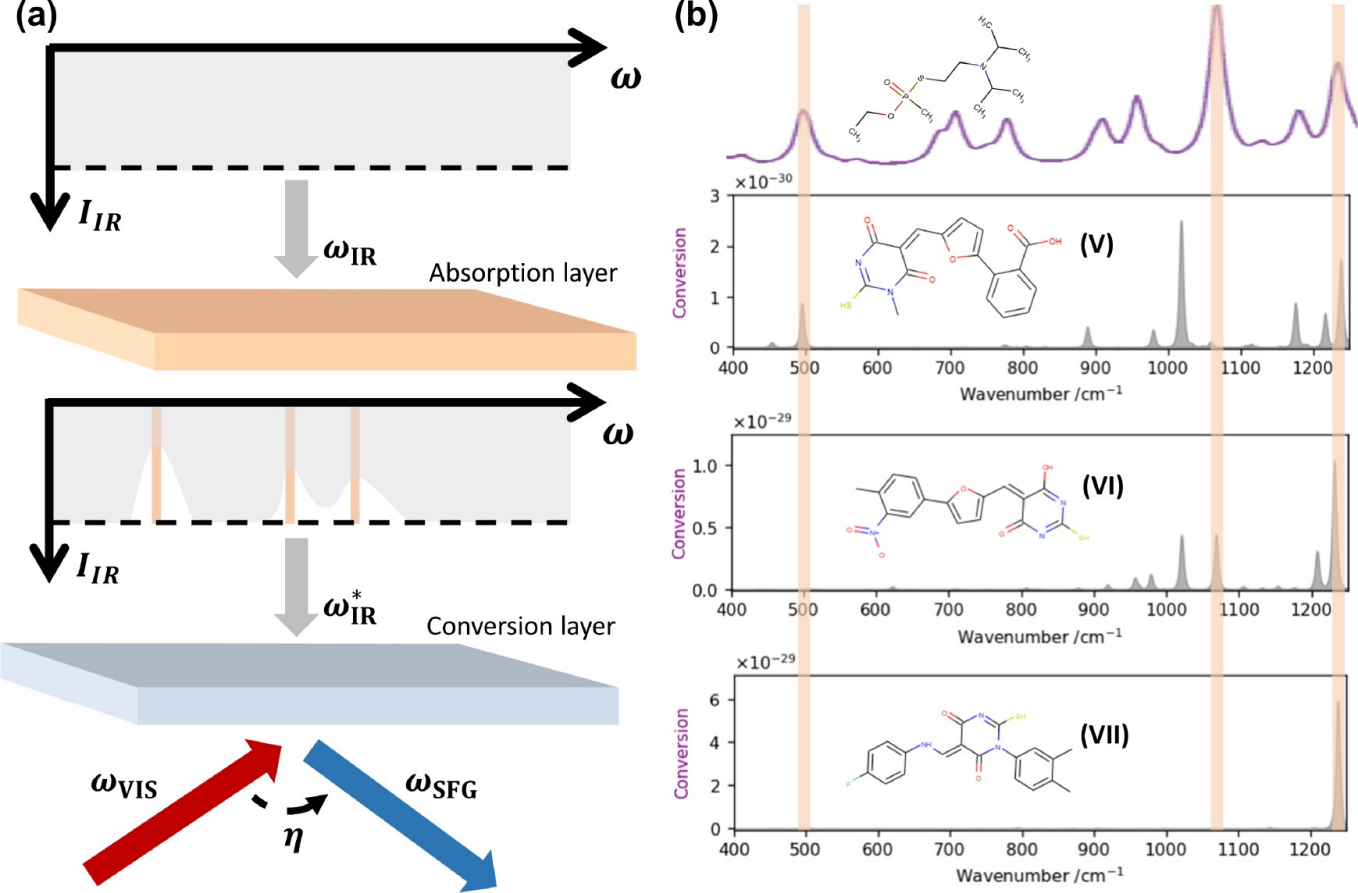
(a) Schematics of the proposed detection scheme for substance identification. From top to bottom, broadband infrared light is passing through the substance to be identified,^34^ after which it is analyzed thanks to an upconversion layer containing molecules with SFG active modes at matched frequencies, so that spectroscopy is eventually performed using visible light detectors. (b) Example of SFG active molecules identified in the database with vibrational frequencies matching the main characteristic IR absorption lines of the VX nerve agent. The top spectrum corresponds to the numerically calculated absorption spectrum of the VX nerve agent (adapted from ref (35)). Then, from top to bottom, the conversion spectra correspond to the spectra extracted from the MVE database for the compounds V, VI, and VII. Full name of the molecules are given in SI, section S4.

One prospective technological application of the database is related to the subwavelength dimension of the newly demonstrated SFG devices.^4^ In advanced designs, constituted of different subwavelength–frequency upconverters, simultaneous detection at different frequencies would be possible, akin to metapixel imaging systems realized with dielectric nanostructures.^34^ Such IR recognition devices could be valuable for the detection of toxic substances. For example, the VX nerve agent has different characteristic IR absorption lines^35^ that could well be targeted by SFG nanodevices. Figure 6 shows different molecules extracted from the database that could efficiently detect variations of IR absorption at the 490, 1067, and 1232 cm^–1^ characteristic lines of the VX nerve agent.

## 4 Conclusions

In conclusion, we presented the Molecular Vibrational Explorer (hosted on the Materials Cloud open platform) that allows interactive access to a large database of vibrational and spectroscopic properties for thousands of molecules. The database targets surface-enhanced applications such as SERS, SEIRA, and vibration-assisted frequency upconversion; it can also be expanded in the future with more molecules. We illustrated the use of the Molecular Vibrational Explorer in the optimization of molecules for a number of applications including SERS tags, vibrational strong coupling, and frequency upconversion for substance detection. We believe that the Molecular Vibrational Explorer will foster progress and productivity in a broad range of research fields related to molecular spectroscopy, nano-optics, and surface science.

## Supporting Information Available

Analytic formulas for the orientation averages, screenshots of the web application pages, example for comparing molecules in “Thiol” and “Gold” databases, chemical name, and SMILES code of molecules (PDF)
