# Supplementary material for: Molecular Vibration Explorer: an Online Database and Toolbox for Surface-Enhanced Frequency Conversion and Infrared and Raman Spectroscopy
Source: J Phys Chem A. 2022 Jul 6;126(28):4657–63. doi: 10.1021/acs.jpca.2c03700 (PMC9310003; doi:10.1021/acs.jpca.2c03700)
Supplement: Supplementary file 1 — jp2c03700_si_001.pdf [file jp2c03700_si_001.pdf]

Supporting Information for Molecular Vibration Explorer: an Online Database and Toolbox for Surface-Enhanced Frequency Conversion, Infrared and Raman Spectroscopy

Zsuzsanna Koczor-Benda,[*,†,‡] Philippe Roelli,[¶] Christophe Galland,[§] and Edina Rosta[†,‡]

†*Department of Physics and Astronomy, University College London, London, WC1E 6BT, United Kingdom*

‡*Department of Chemistry, King's College London, London, SE1 1DB, United Kingdom*

¶*Nano-optics group, CIC nanoGUNE BRTA, 20018 San Sebastián, Spain*

§*Institute of Physics, Ecole Polytechnique Fédérale de Lausanne (EPFL), CH-1015 Lausanne, Switzerland*

E-mail: z.koczor-benda@ucl.ac.uk

S1 Analytic formulas for orientation-averaging

There are four unique integrals for all the possible arrangements of the field vectors, these are given in the following. Subscripts a, b, and c denote field directions orthogonal to each other.

$$
\begin{aligned}
&\langle|\underline{e}_a\,\underline{\mu}'_m|^2|\underline{e}_a\,\underline{\underline{\alpha}}'_m\,\underline{e}_a|^2\rangle = \\
&\frac{1}{105}\big(2\alpha'_{m,\mathrm{xx}}\big(12\mu'_{m,\mathrm{x}}\alpha'_{m,\mathrm{xy}}\mu'_{m,y}+4\mu'_{m,z}\big(3\mu'_{m,\mathrm{x}}\alpha'_{m,\mathrm{xz}}+\mu'_{m,y}\alpha'_{m,\mathrm{yz}}\big)\\
&\quad+\alpha'_{m,\mathrm{yy}}\big(3\big(\mu'_{m,\mathrm{x}}\big)^2+3\big(\mu'_{m,y}\big)^2+\big(\mu'_{m,z}\big)^2\big)\\
&\quad+\alpha'_{m,\mathrm{zz}}\big(3\big(\mu'_{m,\mathrm{x}}\big)^2+\big(\mu'_{m,y}\big)^2+3\big(\mu'_{m,z}\big)^2\big)\big)\\
&\quad+3\big(\alpha'_{m,\mathrm{xx}}\big)^2\big(5\big(\mu'_{m,\mathrm{x}}\big)^2+\big(\mu'_{m,y}\big)^2+\big(\mu'_{m,z}\big)^2\big)\\
&\quad+8\alpha'_{m,\mathrm{xy}}\big(2\mu'_{m,z}\big(\mu'_{m,\mathrm{x}}\alpha'_{m,\mathrm{yz}}+\alpha'_{m,\mathrm{xz}}\mu'_{m,y}\big)\\
&\quad+\mu'_{m,\mathrm{x}}\mu'_{m,y}\big(3\alpha'_{m,\mathrm{yy}}+\alpha'_{m,\mathrm{zz}}\big)\big)\\
&\quad+4\big(\alpha'_{m,\mathrm{xy}}\big)^2\big(3\big(\big(\mu'_{m,\mathrm{x}}\big)^2+\big(\mu'_{m,y}\big)^2\big)+\big(\mu'_{m,z}\big)^2\big)\\
&\quad+12\big(\mu'_{m,\mathrm{x}}\big)^2\big(\alpha'_{m,\mathrm{xz}}\big)^2\\
&\quad+8\mu'_{m,z}\big(\mu'_{m,\mathrm{x}}\alpha'_{m,\mathrm{xz}}\big(\alpha'_{m,\mathrm{yy}}+3\alpha'_{m,\mathrm{zz}}\big)+3\mu'_{m,y}\alpha'_{m,\mathrm{yz}}\big(\alpha'_{m,\mathrm{yy}}+\alpha'_{m,\mathrm{zz}}\big)\big)\\
&\quad+16\mu'_{m,\mathrm{x}}\alpha'_{m,\mathrm{xz}}\mu'_{m,y}\alpha'_{m,\mathrm{yz}}+3\big(\mu'_{m,\mathrm{x}}\big)^2\big(\alpha'_{m,\mathrm{yy}}\big)^2\\
&\quad+2\big(\mu'_{m,\mathrm{x}}\big)^2\alpha'_{m,\mathrm{yy}}\alpha'_{m,\mathrm{zz}}+4\big(\mu'_{m,\mathrm{x}}\big)^2\big(\alpha'_{m,\mathrm{yz}}\big)^2+3\big(\mu'_{m,\mathrm{x}}\big)^2\big(\alpha'_{m,\mathrm{zz}}\big)^2\\
&\quad+4\big(\alpha'_{m,\mathrm{xz}}\big)^2\big(\mu'_{m,y}\big)^2+3\big(\mu'_{m,z}\big)^2\big(4\big(\alpha'_{m,\mathrm{xz}}\big)^2\\
&\quad+\big(\alpha'_{m,\mathrm{yy}}\big)^2+2\alpha'_{m,\mathrm{yy}}\alpha'_{m,\mathrm{zz}}+4\big(\alpha'_{m,\mathrm{yz}}\big)^2+5\big(\alpha'_{m,\mathrm{zz}}\big)^2\big)\\
&\quad+15\big(\mu'_{m,y}\big)^2\big(\alpha'_{m,\mathrm{yy}}\big)^2+6\big(\mu'_{m,y}\big)^2\alpha'_{m,\mathrm{yy}}\alpha'_{m,\mathrm{zz}}\\
&\quad+12\big(\mu'_{m,y}\big)^2\big(\alpha'_{m,\mathrm{yz}}\big)^2+3\big(\mu'_{m,y}\big)^2\big(\alpha'_{m,\mathrm{zz}}\big)^2\big)
\end{aligned}
\tag{S1}
$$

$$
\begin{aligned}
&\langle|\underline{e}_b\,\underline{\mu}'_m|^2|\underline{e}_a\,\underline{\underline{\alpha}}'_m\,\underline{e}_a|^2\rangle = \\
&\frac{1}{105}\Big(3\left(\mu'_{m,\mathrm{x}}\right)^2\left(\alpha'_{m,\mathrm{xx}}\right)^2 \\
&-4\mu'_{m,z}\left(\mu'_{m,\mathrm{x}}\alpha'_{m,\mathrm{xz}}\left(3\alpha'_{m,\mathrm{xx}}+\alpha'_{m,\mathrm{yy}}+3\alpha'_{m,\mathrm{zz}}\right)+2\mu'_{m,\mathrm{x}}\alpha'_{m,\mathrm{xy}}\alpha'_{m,\mathrm{yz}}\right. \\
&\left.+\mu'_{m,y}\alpha'_{m,\mathrm{yz}}\left(\alpha'_{m,\mathrm{xx}}+3\left(\alpha'_{m,\mathrm{yy}}+\alpha'_{m,\mathrm{zz}}\right)\right)+2\alpha'_{m,\mathrm{xy}}\alpha'_{m,\mathrm{xz}}\mu'_{m,y}\right) \\
&-12\mu'_{m,\mathrm{x}}\alpha'_{m,\mathrm{xx}}\alpha'_{m,\mathrm{xy}}\mu'_{m,y}+4\left(\mu'_{m,\mathrm{x}}\right)^2\alpha'_{m,\mathrm{xx}}\alpha'_{m,\mathrm{yy}} \\
&+4\left(\mu'_{m,\mathrm{x}}\right)^2\alpha'_{m,\mathrm{xx}}\alpha'_{m,\mathrm{zz}}+8\left(\mu'_{m,\mathrm{x}}\right)^2\left(\alpha'_{m,\mathrm{xy}}\right)^2 \\
&-12\mu'_{m,\mathrm{x}}\alpha'_{m,\mathrm{xy}}\mu'_{m,y}\alpha'_{m,\mathrm{yy}}-4\mu'_{m,\mathrm{x}}\alpha'_{m,\mathrm{xy}}\mu'_{m,y}\alpha'_{m,\mathrm{zz}} \\
&+8\left(\mu'_{m,\mathrm{x}}\right)^2\left(\alpha'_{m,\mathrm{xz}}\right)^2-8\mu'_{m,\mathrm{x}}\alpha'_{m,\mathrm{xz}}\mu'_{m,y}\alpha'_{m,\mathrm{yz}}+9\left(\mu'_{m,\mathrm{x}}\right)^2\left(\alpha'_{m,\mathrm{yy}}\right)^2 \\
&+6\left(\mu'_{m,\mathrm{x}}\right)^2\alpha'_{m,\mathrm{yy}}\alpha'_{m,\mathrm{zz}}+12\left(\mu'_{m,\mathrm{x}}\right)^2\left(\alpha'_{m,\mathrm{yz}}\right)^2+9\left(\mu'_{m,\mathrm{x}}\right)^2\left(\alpha'_{m,\mathrm{zz}}\right)^2 \\
&+\left(\mu'_{m,z}\right)^2\left(9\left(\alpha'_{m,\mathrm{xx}}\right)^2+6\alpha'_{m,\mathrm{xx}}\alpha'_{m,\mathrm{yy}}+4\alpha'_{m,\mathrm{zz}}\left(\alpha'_{m,\mathrm{xx}}+\alpha'_{m,\mathrm{yy}}\right)\right. \\
&\left.+12\left(\alpha'_{m,\mathrm{xy}}\right)^2+8\left(\alpha'_{m,\mathrm{xz}}\right)^2+9\left(\alpha'_{m,\mathrm{yy}}\right)^2+8\left(\alpha'_{m,\mathrm{yz}}\right)^2+3\left(\alpha'_{m,\mathrm{zz}}\right)^2\right) \\
&+9\left(\alpha'_{m,\mathrm{xx}}\right)^2\left(\mu'_{m,y}\right)^2+4\alpha'_{m,\mathrm{xx}}\left(\mu'_{m,y}\right)^2\alpha'_{m,\mathrm{yy}}+6\alpha'_{m,\mathrm{xx}}\left(\mu'_{m,y}\right)^2\alpha'_{m,\mathrm{zz}} \\
&+8\left(\alpha'_{m,\mathrm{xy}}\right)^2\left(\mu'_{m,y}\right)^2+12\left(\alpha'_{m,\mathrm{xz}}\right)^2\left(\mu'_{m,y}\right)^2+3\left(\mu'_{m,y}\right)^2\left(\alpha'_{m,\mathrm{yy}}\right)^2 \\
&+4\left(\mu'_{m,y}\right)^2\alpha'_{m,\mathrm{yy}}\alpha'_{m,\mathrm{zz}}+8\left(\mu'_{m,y}\right)^2\left(\alpha'_{m,\mathrm{yz}}\right)^2+9\left(\mu'_{m,y}\right)^2\left(\alpha'_{m,\mathrm{zz}}\right)^2\Big)
\end{aligned}
\tag{S2}
$$

$$
\begin{aligned}
&\langle|\underline{e}_a\,\underline{\mu}'_m|^2|\underline{e}_a\,\underline{\underline{\alpha}}'_m\,\underline{e}_b|^2\rangle = \langle|\underline{e}_a\,\underline{\mu}'_m|^2|\underline{e}_b\,\underline{\underline{\alpha}}'_m\,\underline{e}_a|^2\rangle = \\
&\frac{1}{105}\Big(-\alpha'_{m,\mathrm{xx}}\left(-2\left(\mu'_{m,\mathrm{x}}\alpha'_{m,\mathrm{xy}}\mu'_{m,y}+\mu'_{m,z}\left(\mu'_{m,\mathrm{x}}\alpha'_{m,\mathrm{xz}}-2\mu'_{m,y}\alpha'_{m,\mathrm{yz}}\right)\right)\right. \\
&\left.+\alpha'_{m,\mathrm{yy}}\left(3\left(\mu'_{m,\mathrm{x}}\right)^2+3\left(\mu'_{m,y}\right)^2+\left(\mu'_{m,z}\right)^2\right)+\alpha'_{m,\mathrm{zz}}\left(3\left(\mu'_{m,\mathrm{x}}\right)^2+\left(\mu'_{m,y}\right)^2+3\left(\mu'_{m,z}\right)^2\right)\right) \\
&+\left(\alpha'_{m,\mathrm{xx}}\right)^2\left(3\left(\mu'_{m,\mathrm{x}}\right)^2+2\left(\left(\mu'_{m,y}\right)^2+\left(\mu'_{m,z}\right)^2\right)\right)+6\alpha'_{m,\mathrm{xy}}\mu'_{m,z}\left(\mu'_{m,\mathrm{x}}\alpha'_{m,\mathrm{yz}}+\alpha'_{m,\mathrm{xz}}\mu'_{m,y}\right) \\
&+2\mu'_{m,\mathrm{x}}\alpha'_{m,\mathrm{xy}}\mu'_{m,y}\left(\alpha'_{m,\mathrm{yy}}-2\alpha'_{m,\mathrm{zz}}\right)+\left(\alpha'_{m,\mathrm{xy}}\right)^2\left(8\left(\left(\mu'_{m,\mathrm{x}}\right)^2+\left(\mu'_{m,y}\right)^2\right)+5\left(\mu'_{m,z}\right)^2\right) \\
&+8\left(\mu'_{m,\mathrm{x}}\right)^2\left(\alpha'_{m,\mathrm{xz}}\right)^2+2\mu'_{m,z}\left(\mu'_{m,\mathrm{x}}\alpha'_{m,\mathrm{xz}}\left(\alpha'_{m,\mathrm{zz}}-2\alpha'_{m,\mathrm{yy}}\right)+\mu'_{m,y}\alpha'_{m,\mathrm{yz}}\left(\alpha'_{m,\mathrm{yy}}+\alpha'_{m,\mathrm{zz}}\right)\right) \\
&+6\mu'_{m,\mathrm{x}}\alpha'_{m,\mathrm{xz}}\mu'_{m,y}\alpha'_{m,\mathrm{yz}}+2\left(\mu'_{m,\mathrm{x}}\right)^2\left(\alpha'_{m,\mathrm{yy}}\right)^2-\left(\mu'_{m,\mathrm{x}}\right)^2\alpha'_{m,\mathrm{yy}}\alpha'_{m,\mathrm{zz}} \\
&+5\left(\mu'_{m,\mathrm{x}}\right)^2\left(\alpha'_{m,\mathrm{yz}}\right)^2+2\left(\mu'_{m,\mathrm{x}}\right)^2\left(\alpha'_{m,\mathrm{zz}}\right)^2+5\left(\alpha'_{m,\mathrm{xz}}\right)^2\left(\mu'_{m,y}\right)^2 \\
&+\left(\mu'_{m,z}\right)^2\left(8\left(\alpha'_{m,\mathrm{xz}}\right)^2+2\left(\alpha'_{m,\mathrm{yy}}\right)^2-3\alpha'_{m,\mathrm{yy}}\alpha'_{m,\mathrm{zz}}+8\left(\alpha'_{m,\mathrm{yz}}\right)^2+3\left(\alpha'_{m,\mathrm{zz}}\right)^2\right) \\
&+3\left(\mu'_{m,y}\right)^2\left(\alpha'_{m,\mathrm{yy}}\right)^2-3\left(\mu'_{m,y}\right)^2\alpha'_{m,\mathrm{yy}}\alpha'_{m,\mathrm{zz}}+8\left(\mu'_{m,y}\right)^2\left(\alpha'_{m,\mathrm{yz}}\right)^2+2\left(\mu'_{m,y}\right)^2\left(\alpha'_{m,\mathrm{zz}}\right)^2\Big)
\end{aligned}
\tag{S3}
$$

$$\langle|\underline{e}_a\ \underline{\mu}'_m|^2|\underline{e}_b\ \underline{\underline{\alpha}}'_m\ \underline{e}_c|^2\rangle =$$

$$\begin{aligned}
&\frac{1}{105}\Big(-\alpha'_{m,\mathrm{xx}}\left(4\mu'_{m,\mathrm{x}}\alpha'_{m,\mathrm{xy}}\mu'_{m,y}+4\mu'_{m,z}\left(\mu'_{m,\mathrm{x}}\alpha'_{m,\mathrm{xz}}-2\mu'_{m,y}\alpha'_{m,\mathrm{yz}}\right)\right.\\
&+\alpha'_{m,\mathrm{yy}}\left(\left(\mu'_{m,\mathrm{x}}\right)^2+\left(\mu'_{m,y}\right)^2+5\left(\mu'_{m,z}\right)^2\right)\\
&\left.+\alpha'_{m,\mathrm{zz}}\left(\left(\mu'_{m,\mathrm{x}}\right)^2+5\left(\mu'_{m,y}\right)^2+\left(\mu'_{m,z}\right)^2\right)\right)\\
&+\left(\alpha'_{m,\mathrm{xx}}\right)^2\left(\left(\mu'_{m,\mathrm{x}}\right)^2+3\left(\left(\mu'_{m,y}\right)^2+\left(\mu'_{m,z}\right)^2\right)\right)\\
&-4\alpha'_{m,\mathrm{xy}}\left(3\mu'_{m,z}\left(\mu'_{m,\mathrm{x}}\alpha'_{m,\mathrm{yz}}+\alpha'_{m,\mathrm{xz}}\mu'_{m,y}\right)\right.\\
&\left.+\mu'_{m,\mathrm{x}}\mu'_{m,y}\left(\alpha'_{m,\mathrm{yy}}-2\alpha'_{m,\mathrm{zz}}\right)\right)\\
&+\left(\alpha'_{m,\mathrm{xy}}\right)^2\left(5\left(\left(\mu'_{m,\mathrm{x}}\right)^2+\left(\mu'_{m,y}\right)^2\right)+11\left(\mu'_{m,z}\right)^2\right)\\
&+5\left(\mu'_{m,\mathrm{x}}\right)^2\left(\alpha'_{m,\mathrm{xz}}\right)^2-12\mu'_{m,\mathrm{x}}\alpha'_{m,\mathrm{xz}}\mu'_{m,y}\alpha'_{m,\mathrm{yz}}\\
&+4\mu'_{m,\mathrm{x}}\alpha'_{m,\mathrm{xz}}\mu'_{m,z}\left(2\alpha'_{m,\mathrm{yy}}-\alpha'_{m,\mathrm{zz}}\right)+3\left(\mu'_{m,\mathrm{x}}\right)^2\left(\alpha'_{m,\mathrm{yy}}\right)^2\\
&-5\left(\mu'_{m,\mathrm{x}}\right)^2\alpha'_{m,\mathrm{yy}}\alpha'_{m,\mathrm{zz}}+11\left(\mu'_{m,\mathrm{x}}\right)^2\left(\alpha'_{m,\mathrm{yz}}\right)^2\\
&+3\left(\mu'_{m,\mathrm{x}}\right)^2\left(\alpha'_{m,\mathrm{zz}}\right)^2+11\left(\alpha'_{m,\mathrm{xz}}\right)^2\left(\mu'_{m,y}\right)^2+\left(\mu'_{m,z}\right)^2\left(5\left(\alpha'_{m,\mathrm{xz}}\right)^2\right.\\
&\left.+3\left(\alpha'_{m,\mathrm{yy}}\right)^2-\alpha'_{m,\mathrm{yy}}\alpha'_{m,\mathrm{zz}}+5\left(\alpha'_{m,\mathrm{yz}}\right)^2+\left(\alpha'_{m,\mathrm{zz}}\right)^2\right)\\
&+\left(\mu'_{m,y}\right)^2\left(\alpha'_{m,\mathrm{yy}}\right)^2-4\mu'_{m,y}\alpha'_{m,\mathrm{yz}}\mu'_{m,z}\left(\alpha'_{m,\mathrm{yy}}+\alpha'_{m,\mathrm{zz}}\right)\\
&-\left(\mu'_{m,y}\right)^2\alpha'_{m,\mathrm{yy}}\alpha'_{m,\mathrm{zz}}+5\left(\mu'_{m,y}\right)^2\left(\alpha'_{m,\mathrm{yz}}\right)^2+3\left(\mu'_{m,y}\right)^2\left(\alpha'_{m,\mathrm{zz}}\right)^2\Big)
\end{aligned} \tag{S4}$$

S2 Captions of the web application notebooks

Three interactive notebooks are available through our web application called Molecular Vibration Explorer. Their functionalities are described in the main text (Section IV - Functionalities) and screenshots are shown in Figs S1, S2, and S3.

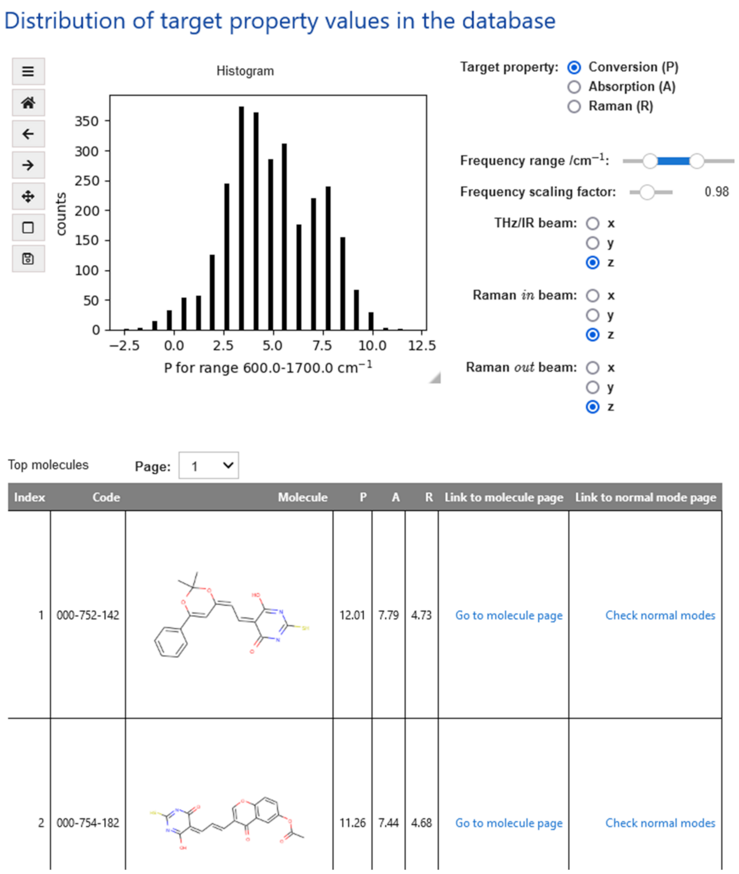

Figure S1: Notebook enabling the exploration of the Gold and Thiol databases. Accessed June 9, 2022.

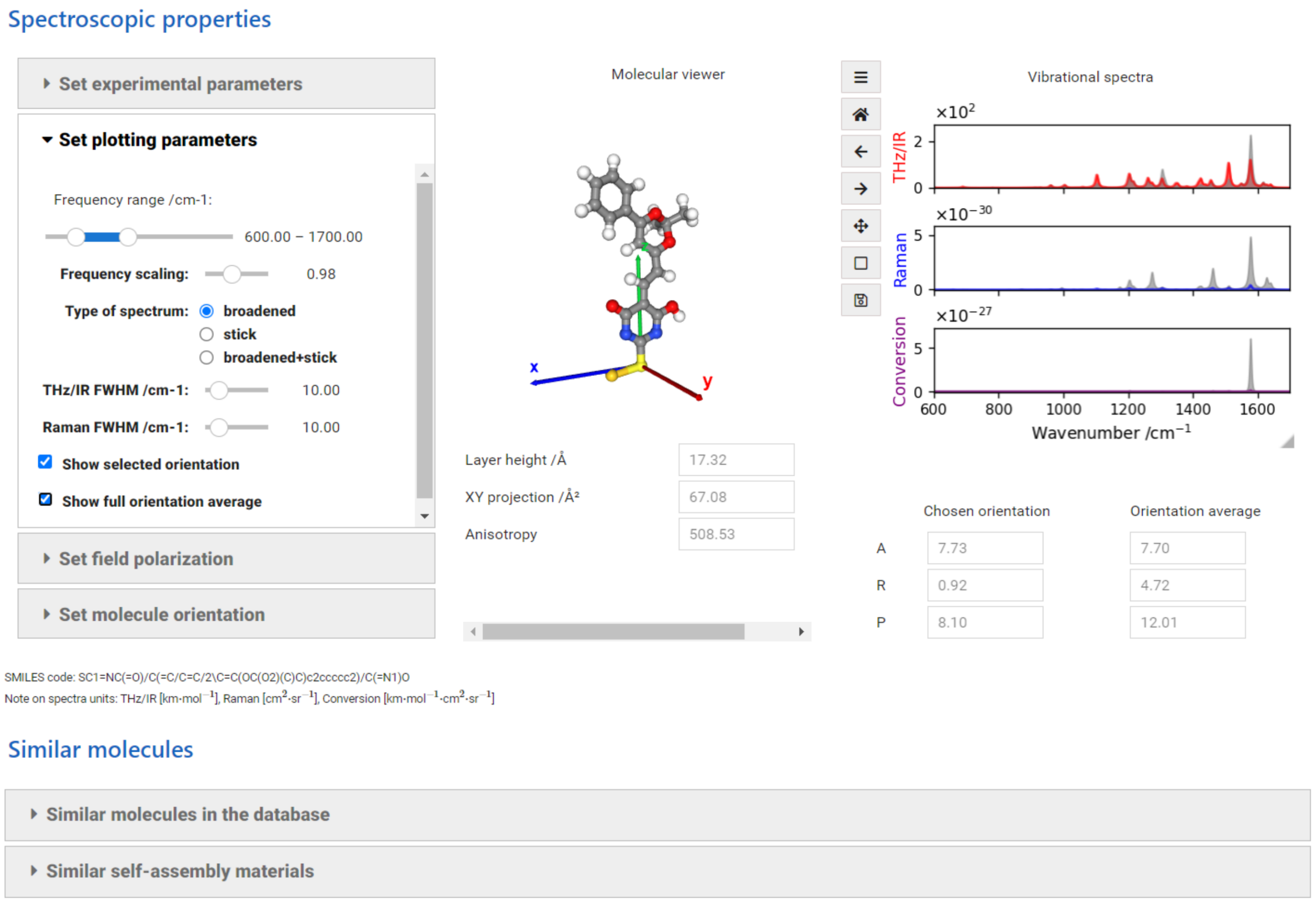

Figure S2: Notebook displaying the molecular properties and vibrational spectra of a single molecule. Accessed June 9, 2022.

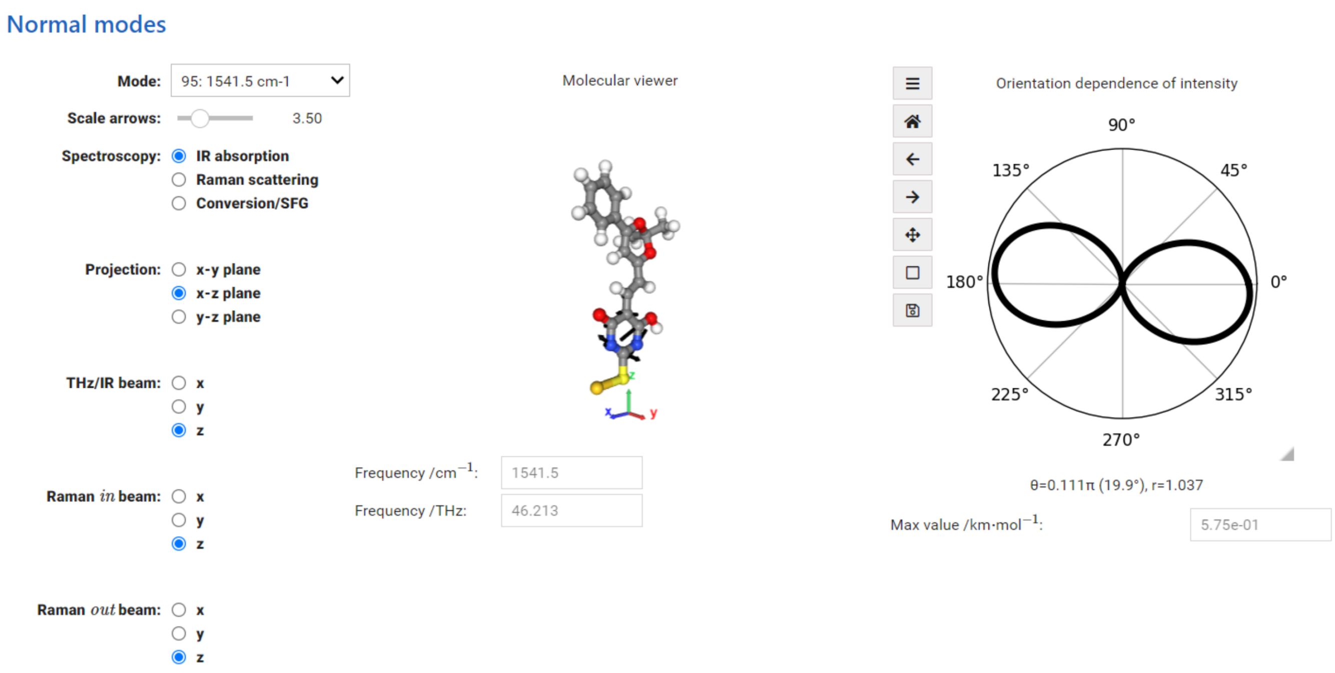

Figure S3: Notebook for the exploration of the different normal modes of the selected molecule. Accessed June 9, 2022.

S3 Comparing the *Thiol* and the *Gold* database

Thiolated and gold-linked versions of a molecule can be compared by a simple SMILES search and navigating the corresponding pages. The example in Fig. S4 shows orientation-averaged spectra and two vibrational modes, one enhanced considerably by linkage to a gold atom, and one that is active in both versions of the molecule.

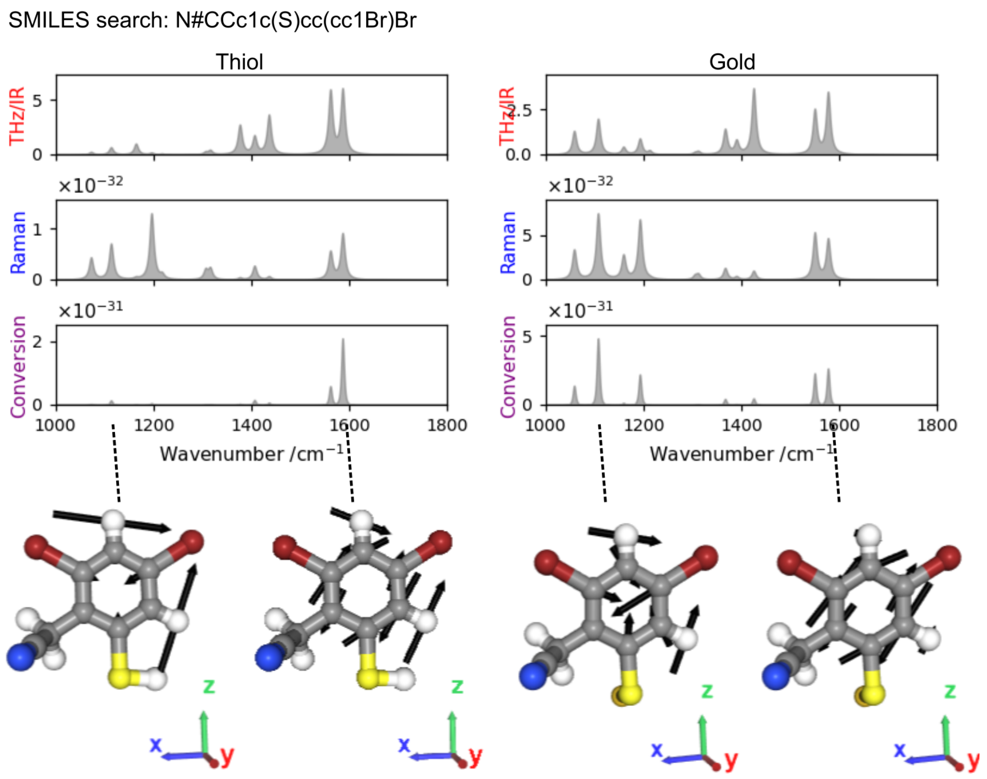

Figure S4: Using MVE to compare thiolated and gold-linked versions of a molecule.

Fig. S5 shows a comparison to experimental SERS and solution phase Raman data from Ref. 1.

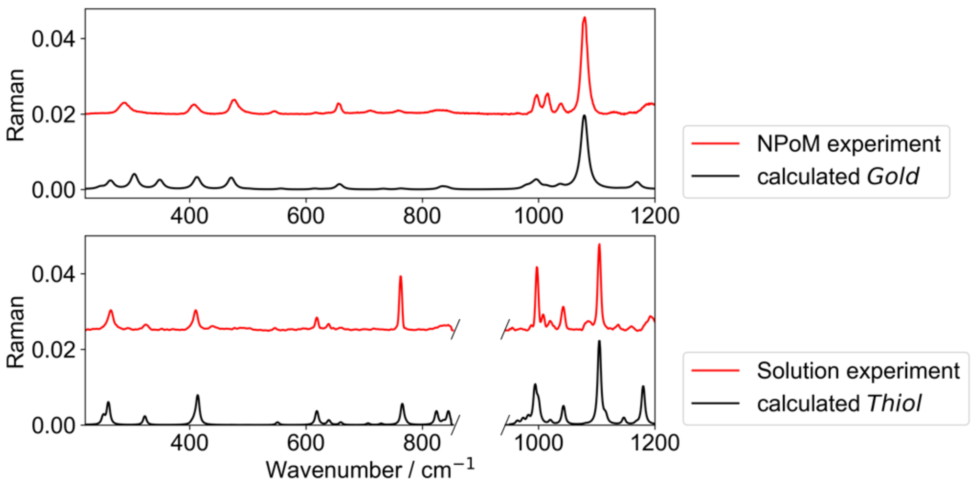

Figure S5: Raman spectrum of BPhT from SERS nanoparticle-on-mirror (NPoM) and solution (solvent: THF) measurements compared to calculations from the *Gold* and *Thiol* databases, respectively. Experimental data was taken from Ref. 1. Intensities are normalized for the integral of the spectra.

S4 List of molecules appearing in the manuscript

Table S1: Full names and SMILES of molecules appearing in the manuscript.

Name manuscript	IUPAC name	SMILES
NH2-BPhT	4-(4-aminophenyl)benzenethiol	C1=CC(=CC=C1C2=CC=C(C=C2)S)N
BPhT	[1,1'-biphenyl]-4-thiol	Sc1ccc(cc1)-c1ccccc1
Br-BPhT	4-(4-bromophenyl)benzenethiol	C1=CC(=CC=C1C2=CC=C(C=C2)Br)S
F-BPhT	4'-fluoro-[1,1'-biphenyl]-4-thiol	Fc1ccc(cc1)-c1ccc(S)cc1
Compound I	(4E)-4-{[4-(dimethylamino)phenyl]methylidene}-1-phenyl-2-sulfanyl-4,5-dihydro-1H-imidazol-5-one	CN(C)c1ccc(\C=C2\N=C(S)N(C2=O)c2ccccc2)cc1 |t:9|
Compound II	(5E)-5-{2-[(4E)-2,2-dimethyl-6-phenyl-2,4-dihydro-1,3-dioxin-4-ylidene]ethylidene}-6-hydroxy-2-sulfanyl-4,5-dihydropyrimidin-4-one	CC1(C)O\C(=C\C=C2/C(O)=NC(S)=NC2=O)C=C(O1)c1ccccc1 |c:9,12,17|
Compound III	N'-{[(5Z)-6-hydroxy-4-oxo-2-sulfanyl-4,5-dihydropyrimidin-5-ylidene]methyl}-N-(4,6,7-trimethylquinazolin-2-yl)guanidine	Cc1cc2nc(NC(=N)N\C=C3\C(O)=NC(S)=NC3=O)nc(C)c2cc1C |c:13,16|
Compound IV	(5E)-6-hydroxy-5-{[5-(phenylsulfanyl)furan-2-yl]methylidene}-2-sulfanyl-4,5-dihydropyrimidin-4-one	OC1=NC(S)=NC(=O)\C1=C\c1ccc(Sc2ccccc2)o1 |c:4,t:1|
CBP	9-[4'-(9H-carbazol-9-yl)-[1,1'-biphenyl]-4-yl]-9H-carbazole	c1ccc2c(c1)n(-c1ccc(cc1)-c1ccc(cc1)-n1c3ccccc3c3ccccc13)c1ccccc21
VX nerve agent	N-[2-[ethoxy(methyl)phosphoryl]sulfanylethyl]-N-propan-2-ylpropan-2-amine	CCOP(=O)(C)SCCN(C(C)C)C(C)C
Compound V	2-(5-{[(5Z)-1-methyl-4,6-dioxo-2-sulfanyl-1,4,5,6-tetrahydropyrimidin-5-ylidene]methyl}furan-2-yl)benzoic acid	CN1C(S)=NC(=O)\C(=C\c2ccc(o2)-c2ccccc2C(O)=O)C1=O |c:3|
Compound VI	(5Z)-6-hydroxy-5-{[5-(4-methyl-3-nitrophenyl)furan-2-yl]methylidene}-2-sulfanyl-4,5-dihydropyrimidin-4-one	Cc1ccc(cc1[N+]([O-])=O)-c1ccc(\C=C2\C(O)=NC(S)=NC2=O)o1 |c:18,21|
Compound VII	(5E)-1-(3,4-dimethylphenyl)-5-{[(4-fluorophenyl)amino]methylidene}-2-sulfanyl-1,4,5,6-tetrahydropyrimidine-4,6-dione	Cc1ccc(cc1C)N1C(S)=NC(=O)\C(=C/Nc2ccc(F)cc2)C1=O |c:11|

References

(1) Koczor-Benda, Z.; Boehmke, A. L.; Xomalis, A.; Arul, R.; Readman, C.; Baumberg, J. J.; Rosta, E. Molecular Screening for Terahertz Detection with Machine-Learning-Based Methods. *Phys. Rev. X* **2021**, *11*, 041035.
